# New locus underlying auriculocondylar syndrome (ARCND): 430 kb duplication involving *TWIST1* regulatory elements

**DOI:** 10.1136/jmedgenet-2021-107825

**Published:** 2021-11-08

**Authors:** Vanessa Luiza Romanelli Tavares, Sofia Ligia Guimarães-Ramos, Yan Zhou, Cibele Masotti, Suzana Ezquina, Danielle de Paula Moreira, Henk Buermans, Renato S Freitas, Johan T Den Dunnen, Stephen R F Twigg, Maria Rita Passos-Bueno

**Affiliations:** 1 Genética e Biologia Evolutiva, Universidade de São Paulo Instituto de Biociências, Sao Paulo, Brazil; 2 Clinical Genetics Group, MRC Weatherall Institute of Molecular Medicine, John Radcliffe Hospital, University of Oxford, Oxford, UK; 3 Molecular Oncology Center, Hospital Sírio-Libanês, Sao Paulo, Brazil; 4 Centre for Cancer Genetic Epidemiology, University of Cambridge, Cambridge, UK; 5 Leiden Genome Technology Center, Leiden University Medical Center, Leiden, The Netherlands; 6 Centro de Atendimento Integral ao Fissurado Lábio Palatal, Curitiba, Brazil

**Keywords:** gene duplication, congenital, hereditary, and neonatal diseases and abnormalities, genetic variation, high-throughput nucleotide sequencing, human genetics

## Abstract

**Background:**

Auriculocondylar syndrome (ARCND) is a rare genetic disease that affects structures derived from the first and second pharyngeal arches, mainly resulting in micrognathia and auricular malformations. To date, pathogenic variants have been identified in three genes involved in the EDN1-DLX5/6 pathway (*PLCB4*, *GNAI3* and *EDN1*) and some cases remain unsolved. Here we studied a large unsolved four-generation family.

**Methods:**

We performed linkage analysis, resequencing and Capture-C to investigate the causative variant of this family. To test the pathogenicity of the CNV found, we modelled the disease in patient craniofacial progenitor cells, including induced pluripotent cell (iPSC)-derived neural crest and mesenchymal cells.

**Results:**

This study highlights a fourth locus causative of ARCND, represented by a tandem duplication of 430 kb in a candidate region on chromosome 7 defined by linkage analysis. This duplication segregates with the disease in the family (LOD score=2.88) and includes *HDAC9*, which is located over 200 kb telomeric to the top candidate gene *TWIST1*. Notably, Capture-C analysis revealed multiple cis interactions between the *TWIST1* promoter and possible regulatory elements within the duplicated region. Modelling of the disease revealed an increased expression of *HDAC9* and its neighbouring gene, *TWIST1*, in neural crest cells. We also identified decreased migration of iPSC-derived neural crest cells together with dysregulation of osteogenic differentiation in iPSC-affected mesenchymal stem cells.

**Conclusion:**

Our findings support the hypothesis that the 430 kb duplication is causative of the ARCND phenotype in this family and that deregulation of *TWIST1* expression during craniofacial development can contribute to the phenotype.

## Introduction

Auriculocondylar syndrome (ARCND) (OMIM #602483, #614669 and #615706), also referred to as ‘question mark ear syndrome’, is a rare Mendelian disorder with a prevalence of under 1 in 1 000 000 (Orphanet; http://www.orpha.net/consor/cgi-bin/index.php). ARCND is characterised by micrognathia, question mark ears, mandibular condyle hypoplasia, and other less common features such as microstomia, glossoptosis, postauricular tags and prominent cheeks.^1 2^ There is wide clinical variability, including cases with isolated ear anomalies. Treatment is mainly corrective through surgical intervention for mandibular ramus lengthening using distraction osteogenesis and accompanied by orthodontic treatments and speech therapy.^3^ Understanding the aetiology of this disorder and elucidating genetic causes improve counselling and may lead to the development of preventive or therapeutic strategies, besides deepening our knowledge of craniofacial development.

The main structures affected in ARCND are derived from the first and second pharyngeal arches that are colonised by neural crest cells (NCCs), originating from the neural plate border by epithelial mesenchymal transition, and mesenchymal stem cells. NCCs are multipotent cells with high migratory ability that can differentiate into several derivatives such as cartilage, bone, peripheral neurons, melanocytes and glia, and they have a central role in craniofacial development.^4^ Disruption in these migratory, patterning or differentiation processes may result in congenital craniofacial malformations.^5^ So far, pathogenic variants in patients with ARCND have been found in genes of the EDN1-endothelin-1 receptor type A (EDNRA) pathway, which are expressed by the neural crest-derived ectomesenchymal cells of pharyngeal arches and are responsible for the patterning of the mandibular domain in the first arch.^6–8^ The most commonly mutated gene in individuals with ARCND is *PLCB4* (MIM 600810; 58% of patients), followed by *GNAI3* (MIM 139370; 19% of cases) and *EDN1* (MIM 131240 and MIM 139370; 15% of cases).^9–12^ Approximately 8% of ARCND cases remain unsolved.^9 13^

Most of the knowledge concerning ARCND pathogenesis comes from functional studies using animal models of craniofacial development, such as mouse and zebrafish.^14 15^ However, animal models may not completely reflect what happens in human development,^16^ and human stem cells or induced pluripotent cells (iPSCs) represent a complementary model system to study development in a human-specific context.^17–19^ iPSCs, which can differentiate into cell types such as NCCs and NCC-derived mesenchymal-like stem cells, can provide insight into human craniofacial development where facial structures derived from the first pharyngeal arch are compromised, as successfully exemplified in the case of Richieri-Costa-Pereira syndrome (RCPS^17^). RCPS, caused by biallelic, hypomorphic alleles at the DEAD-box helicase *EIF4A3*,^20^ shares overlapping clinical features with ARCND particularly in mandible underdevelopment.^17 20^ In this work, we studied a previously reported ARCND family^13^ and performed genetic and functional investigations using patient iPSCs that had been differentiated into NCC (iPSC-derived NCCs) and mesenchymal-like stem cells (nMSC) derivatives. Our findings suggest that duplication of sequences at the *HDAC9* locus can lead to the development of ARCND, possibly by disruption of regulatory elements that control expression of the neighbouring *TWIST1* gene.

## Materials and methods

### Patients and DNA samples

The Brazilian family (referred to as F1; online supplemental table 1) is a non-consanguineous family with 11 members showing the typical characteristics of ARCND. Of those documented, the majority presented with question mark ears (8 of 10), microstomia (8 of 10) and micrognathia (6 of 10), with considerable intrafamilial variability observed.^13^ Genomic DNA was extracted from peripheral blood lymphocytes according to Miller *et al*.^21^

10.1136/jmedgenet-2021-107825.supp1 Supplementary data

### Sanger sequencing

Primers and conditions used in PCR amplification prior to Sanger sequencing were as described in Romanelli Tavares *et al*.^22^ The data were analysed using Sequencher V.5.1 software (http://genecodes.com/). Variants present in the 1000 Genomes Database, dbSNP150 (through University of California, Santa Cruz Genome Browser, UCSC; https://genome.ucsc.edu/), Genome Aggregation Database (https://gnomad.broadinstitute.org/) or in the Online Archive of Brazilian Mutations (http://abraom.ib.usp.br/) were considered unlikely to be pathogenic.

### Linkage analysis

Nine affected individuals (II-4, II-6, II-8, III-5, III-10, III-13, III-14, IV-3 and IV-6) and three unaffected individuals (III-6, III-11 and IV-4) were genotyped using the GeneChip Human Mapping 50K Array Xba 240 (Affymetrix), according to the manufacturer’s protocol. The genotype data were analysed using Affymetrix Genotyping Console. The overall quality of the samples was estimated through quality control (QC) algorithm (dynamic model algorithm with QC call rate) using a threshold of 90%.

Linkage analysis was performed with the easyLINKAGE-Plus V.5.08 package.^23^ Mendelian inconsistencies were removed using PedCheck V.1.0^24^ and the non-Mendelian inconsistencies with Merlin V.1.0.1 software.^25^ The logarithmic odds (LOD) score was obtained using the parametric multipoint test with GeneHunter V.2.1r5.^23 26^ Analysis parameters were defined as autosomal dominant, estimated penetrance K=0.9, disease allele frequency estimated at 0.0001 and marker spacing at 0.0010 cM, and map distances were acquired from AFFY 100K deCODE Human GRCh37/hg19, and SNPs with a call rate less than 90% were removed.

The candidate region was confirmed by amplification of microsatellite markers from the ABI PRISM Linkage Mapping Set V.2.0 (Perkin-Elmer, Applied Biosystems, Foster City, California), read in a MegaBACE 1000 automatic sequencer (Amersham, GE Healthcare, Little Chalfont, UK) according to the manufacturer’s protocol and analysed with MegaBACE Genetic Profiler software (Amersham, GE Healthcare).

Endeavour gene prioritisation was applied to the candidate Chr7 region (https://endeavour.esat.kuleuven.be/)^27^ to generate a candidate gene list. Training gene lists (reference genes) were compiled according to the following criteria: (a) genes with a central role in the formation of the structures affected in patients with ARCND (eg, ears, mandible and mandibular condyle); or (b) genes related to the embryonic developmental processes involved in the formation of some of the structures affected in patients with ARCND (eg, neural crest and formation of the first and second pharyngeal arches) (online supplemental table 2).

### Whole exome sequencing

Whole exome libraries were generated using either the Agilent SureSelect Human All Exon 50 Mb Kit (patients IV-3 and IV-6; performed at the Center for Human and Clinical Genetics, Leiden University Medical Center, The Netherlands) or the Illumina TruSeq Kit (patients III-10 and III-13; performed at Luiz de Queiroz College of Agriculture, São Paulo, Piracicaba, Brazil). Whole exome sequencing (WES) was carried out on Illumina HiSeq 2000 (2 x 100 bp paired-end run). Sequences were aligned to the human reference GRCh37 (hg19) using the Burrows-Wheeler Aligner (BWA).^28^ Processing and variant calling were performed along with batch samples using the Unified Genotyper tool (Genome Analysis Toolkit, GATK) (http://www.broadinstitute.org/gatk/),^29^ using default parameters, with exception to the following changes: minIndelCnt 3; minIndelFrac 0.020; contamination 0.02; metrics snps.metrics; stand_call_conf 30.0; stand_emit_conf 10.0; min_base_quality_score 12; dcov 300; baq CALCULATE_AS_NECESSARY. Annotation was done with Annovar (http://annovar.openbioinformatics.org/).^30^ Variants were selected if they had been approved by the filter quality (PASS), frequency ≤0.01 in 1000 Genomes Database (https://www.internationalgenome.org/) and Exome Variant Server (ESP6500; https://evs.gs.washington.edu/EVS/), heterozygous genotype in all four affected individuals sequenced, and with allele count ≤4 in the local sequenced cohort of patients without ARCND (total allele number=132).

### Targeted sequencing

Targeted sequencing at the *HDAC9/TWIST1* locus was performed using a resequencing capture panel designed to the *TWIST1* gene and flanking regions (2.4 Mb with boundaries selected according to human to mouse synteny; chr7:17,346,143–19,695,462, GRCh38).^31^ Genomic DNA from family members (three affected and one unaffected family members: IV-3, IV-6, III-5, II-7) was fragmented by sonication, ligated to indexed Illumina sequencing adapters and amplified. Purified libraries were mixed with the biotinylated probe mixture (SeqCap EZ Choice Library System, Roche Nimblegen) and enriched DNA for the targeted regions sequenced on an Illumina HiSeq 2500. Sequencing adapter sequences and low-quality bases were removed using Trimmomatic (V.0.32; parameter SLIDINGWINDOW: 4:20^32^) and the trimmed read pairs were then aligned to human reference genome hg19 using BWA (V.0.7.12) with default parameters.^28^ The aligned reads were analysed using amplimap (V.0.2.9)^33^ and coverage calculated using BEDtools V.0.25.0.^34^ Variants were called separately in each sample using Platypus (V.0.8.1)^35^ and then concatenated, merged and normalised using BCFtools (V.1.5; https://github.com/samtools/bcftools) and annotated with Annovar.^30^

For the breakpoint isolation, we examined the resequencing data at the duplication junctions and designed the following primers to amplify the breakpoint: F-5′-CCCATGCCTCATTCTTTCTTTG-3′ and R-5′-TGGCAGGCTTTAGTGTTCTT-3′.

### Capture-C

To identify the chromatin regions that the *TWIST1* promoter interacts with, we used a Capture-C approach.^36^ For the chromatin template we prepared human mesenchymal cells from human embryonic calvaria (three different samples at 12–14 postconception weeks, provided by the Human Developmental Biology Resource, UK). We removed the skin and dura mater and then dissected a bony strip (approximately 0.5 mm wide) that included the frontal bone, coronal suture and parietal bone and placed this in a gelatinised culture dish containing the following selective medium: BHK-21 Glasgow MEM (Gibco 21710-025)—to this 500 mL we added glutamine (Gibco 25030-024), 2 mM sodium pyruvate (Gibco 11360-039), 100 U/mL penicillin/streptomycin, non-essential amino acids (Gibco 11140-035), 10% Fetal Bovine Serum (FBS) (Gibco 10270), 0.1 mM β-mercaptoethanol and Lif (inhouse prepared Lif-containing medium from modified Chinese Hamster Ovary (CHO) cells). Cells were allowed to grow out of the bone for 3–5 days and then collected (discarding the bony strips) and cultured again using the same media. Cells were passaged at least twice more before collection for 3C library preparation. Cells (10–15 million) were fixed in formaldehyde and then lysed prior to digestion of the cross-linked DNA template with DpnII and DNA ligation. Following DNA purification, the 3C library was sonicated and used to prepare a sequencing library which was then mixed with biotinylated oligonucleotides to enrich for fragments containing the *TWIST1* promoter. Two successive rounds of capture were performed. Biotinylated oligonucleotides were designed using an online tool (http://apps.molbiol.ox.ac.uk/CaptureC/cgibin/CapSequm.cgi) to each side of a DpnII fragment that overlapped with the *TWIST1* promoter: *TWIST1*pro1: ATCCAGTGGACAATTAGGCTTCGTGAGCCCCAATTCCAAATGCTTGGATACGCTAACATTTTAAGCATTTCTGTCTGTAAGTTAAAACGAAGAGCCCCAAAGAGGGTGTTAATGTAGATC and *TWIST1*pro2: GATCTTCCGCAGCGCGGCGAACGCCTCGTTCAGCGACTGGGTGCGCTGGCGCTCCCGCACGTTGGCCATGACCCGCTGCGTCTGCAGCTCCTCGTAAGACTGCGGACTCCCGCCGCCGCT. Captured fragments were sequenced on an Illumina MiSeq (2 x 150 bp paired-end run; MRC Weatherall Institute of Molecular Medicine (WIMM), Oxford).

### Generation of iPSC, NCC and MSC

Three ARCND samples and three control samples were used for generation of iPSCs. One of the control iPSCs used in this study (F7405-1) had been generated with retroviral transduction and was described and characterised elsewhere.^37^ The other cells were established from erythroblast cultures derived from peripheral blood collection (from three affected individuals: II-4, II-8 and III-5; and two non-related controls: F8799 and F9048), reprogrammed as described in Okita *et al* 38 in an Amaxa Nucleofector II (T-016 program for erythroblasts) with either NHDF (Normal Human Dermal Fibroblasts) or CD34+ (erythroblasts) nucleofector kits (Lonza), according to the manufacturer’s recommendations. After nucleoporation, iPSCs were obtained exactly as described in Miller *et al*.^17^ Derivation of NCC from iPSC and Mesenchymal Stem Cells (MSC) differentiation from NCC were also performed as previously published.^39^ Characterisation of iPSC, NCC and MSC is described in the online supplemental material along with the antibodies used (online supplemental table 3). To assess EDN1/EDNRA pathway-related gene expression, NCCs were treated with EDN1 100 nM for 24 hours.

### MSC osteogenic differentiation

Cells were seeded in 12-well plates (Corning) (10^4^ cells/cm^2^) in triplicate. After 3 days, the medium was replaced with an osteogenic induction medium (StemPro Osteogenesis Kit, Life Technologies); in parallel, negative controls were cultivated in MSC medium. Differentiation and the MSC media were changed every 2–3 days. After 9 days, alkaline phosphatase (ALP) activity was quantified through incubation with phosphatase substrate (Sigma-Aldrich), and the resulting *p*-nitrophenol was quantified colourimetrically using Multiskan EX ELISA Plate Reader (Thermo Scientific) at 405 nm. Absorbance data were normalised by subtracting from undifferentiated, negative controls.

### Wound healing assay

NCCs were seeded at 5×10^5^ cells/cm^2^ into non-coated 24-well plates (Corning) in NCC medium. When cells reached 90%–100% confluence, the monolayer was scratched in a straight line with a p200 pipette tip. The culture medium was then replaced and cell migration images were acquired at 0 hour and 24 hours. All samples were assessed simultaneously in two independent experiments. The percentage of the wound covered by migrating cells after 24 hours was quantified in ARCND and control NCCs using ImageJ.

### Cell cycle assay

To determine the percentage of cells in G0/G1, S and G2/M phases based on DNA content, a cell cycle assay was performed using the Guava Cell Cycle Reagent (Millipore). Cells were seeded at a density of 0.2×10^5^ cells/cm^2^. When the cell culture reached 50% confluence, cells were cultured in NCC medium without basic-fibroblast growth factor (bFGF) for 24 hours. Complete NCC medium was added afterwards and the next day cells were detached using Accutase to obtain a single-cell suspension and neutralised with Dulbecco's Modified Eagle Medium (DMEM). Suspended cells were collected in a tube and centrifuged at 450 *g* for 5 min. The supernatant was removed and ice-cold 70% ethanol was added gently to the cell pellet and stored in −20°C for at least 3 hours. Fixed cells were washed in phosphate-buffered saline (PBS), resuspended with Guava Cell Cycle Reagent and incubated for 30 min in the dark. Cells were analysed with the Guava EasyCyte Flow Cytometer (Millipore) according to the manufacturer’s instructions.

### Real-time QPCR

Total RNA was extracted from cells with the NucleoSpin RNA II Extraction Kit (Macherey-Nagel) following the manufacturer’s recommendations. Total RNA was converted into cDNA using SuperScript IV (Life Technologies) and oligo-dT primers. Real-time QPCR reactions were performed with 2X Fast SYBR Green PCR Master Mix (Life Technologies) and 50–400 nM of each primer. Fluorescence was detected using the QuantStudio 5 System (Life Technologies) under a standard temperature protocol. Primer pairs were either designed with Primer-BLAST or retrieved from PrimerBank and supplied by Exxtend (online supplemental table 4). geNorm (https://genorm.cmgg.be/) was used to determine the normalisation factor (using gene expression of TATA-box binding protein (TBP), hydroxymethylbilane synthase (HMBS) or glyceraldehyde-3-phosphate dehydrogenase (GAPDH)) and calculate normalisation factors (E−ΔC) for each sample. The final relative expression values were determined based on the Pfaffl^40^ method.

### Statistical analysis

All experiments were performed in triplicate, unless stated otherwise. Statistical comparisons were performed using GraphPad Prism V.5 software. Unpaired Student’s t-test and two-way analysis of variance (ANOVA) values were represented as mean±SE. The level of statistical significance was set at p<0.05.

## Results

### Evidence of a fourth locus for ARCND

Sanger sequencing of *PLCB4, GNAI3* and *EDN1* did not reveal any pathogenic variants in the coding regions, 5′ UTRs (untranslated regions) or splice sites of these genes, suggesting that a different locus might underlie the ARCND in the family. Next, we carried out a linkage analysis that revealed three regions with positive LOD scores on chromosomes 7, 14 and 18 (online supplemental figure 1). The highest LOD score (2.88), which is close to the threshold value of ≥3.0 for significance^41^ and the maximum theoretical LOD score for this family (2.93), was observed in a region of about 17.6 Mb on chromosome 7 (chr7:14395902–32017194 (hg38); table 1). Genotyping of microsatellite markers narrowed the chromosome 7 linkage region to chr7:14395902–28158440 (hg38) (online supplemental figure 2).

**Table 1:** Regions with positive logarithmic odds (LOD) scores obtained by linkage analysis

Positive LOD score regions							
Chromosome	Maximum LOD score	From		To		Region size (Mb)	Number of genes (NCBI RefSeq curated)
		rsID	Physical position hg38 (bp)	rsID	Physical position hg38 (bp)		
Chr7	2.88	rs1036140	14 395 902	rs28190	32 017 194	17.6	146
Chr14	2.41	rs10484206	49 103 955	rs10498419	49 978 179	0.87	14
Chr18	1.67	rs1398193	48 460 768	rs768360	50 237 703	1.8	20

The maximum theoretical LOD score for this family was equal to 2.93.

We performed WES on four affected individuals. After filtering (as described in the methods), only one variant from the candidate regions on chromosomes 7, 14 and 18 remained, a synonymous change located in *TRIL* (NM_014817.3:c.345G>A; p.(=)), classified as a variant of uncertain significance (BP4, PM2 and PP4 according to the American College of Medical Genetics and Genomics guidelines^42^). Sanger sequencing of additional family members (nine affected and five non-affected individuals) showed that the variant did not segregate with the disease and therefore it was not considered further. Variants in candidate genes within the endothelin pathway (*EDN1*, *EDNRA*, *DLX5*, *DLX6*, *FURIN* and *ECE1*) were also investigated with WES in the same manner, but no obvious pathogenic variants were detected. We then took a gene prioritisation approach, and *TWIST1* was the top-ranked gene in this analysis using two training lists, with a p value equal to 0.00053 and 0.00027 (using training lists ‘a’ and ‘b’, respectively; online supplemental table 5). TWIST1, a basic transcription factor of the helix-loop-helix (bHLH) family, is expressed in cranial mesoderm and neural crest-derived mesenchyme, which are tissues involved in craniofacial development.^43 44^ Its role in mandibular condyle and mandible formation has also been demonstrated.^45 46^ These observations led us to further investigate the genomic region surrounding *TWIST1*.

**Figure 1:**
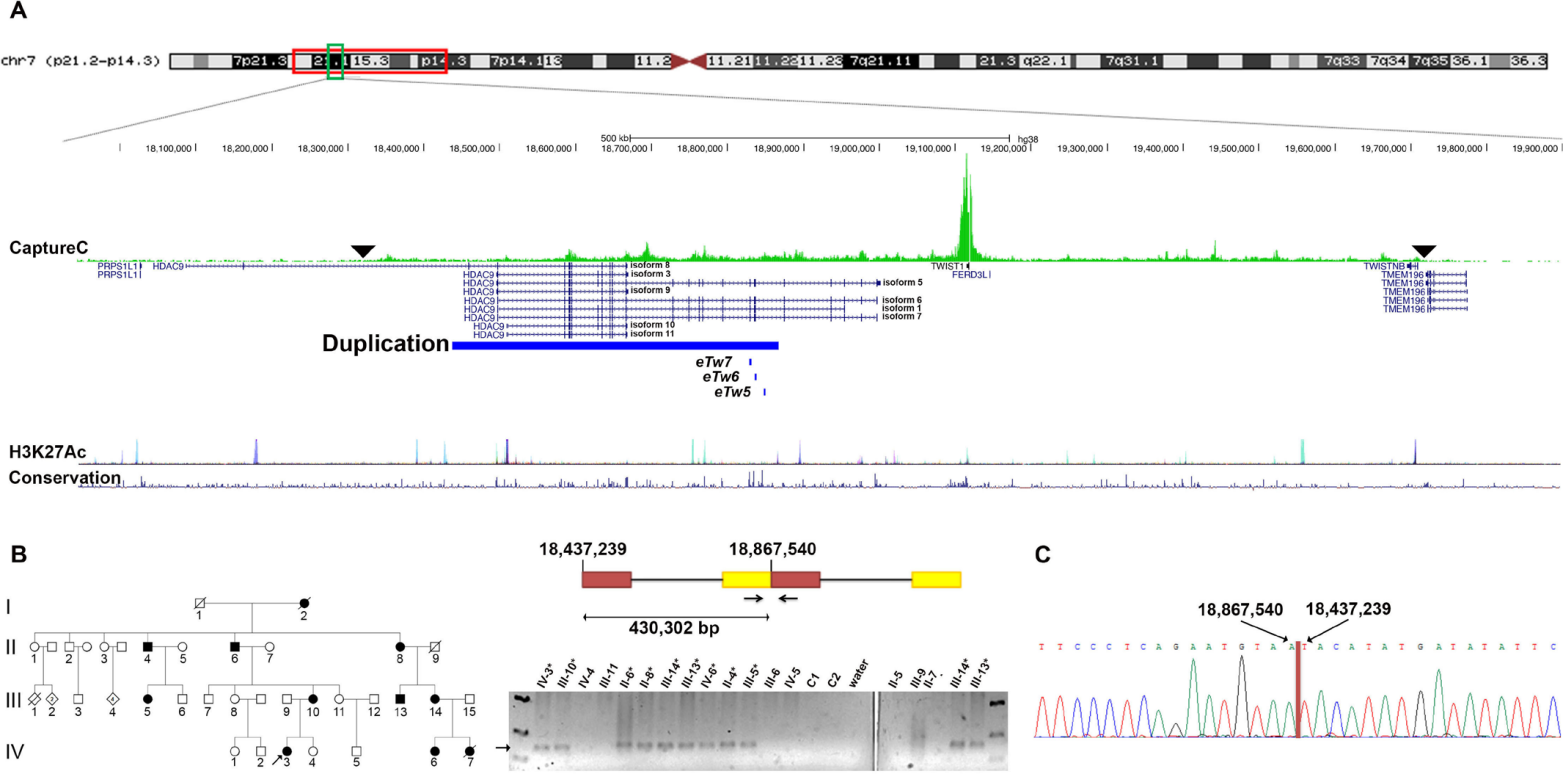
The ARCND 430 kb duplication. (A) Top: ideogram of the chromosome 7 linkage region (red square) indicating the duplicated region (green square). Middle: the *HDAC9/TWIST1* locus and duplicated region (chr7:18 437 238–18 867 540, hg38; blue bar), with Capture-C data above showing cis interactions (the green peaks indicating the frequency of contacts) between the *TWIST1* promoter and possible regulatory elements. The overall domain of interactions is indicated by the black arrowheads; the highest frequency of contacts is within *HDAC9*. The positions of *Twist1* enhancers eTw5-7^51^ are shown in relation to the duplication. Bottom: University of California, Santa Cruz (UCSC) Genome Browser tracks for enrichment levels of the H3K27Ac histone mark across the selected region and conservation (https://genome.ucsc.edu/index.html). (B) Left: pedigree of the ARCND family with the proband indicated. Right: schematic figure of the duplication (NC_000007.14:g.18437239_18867540dup) and breakpoint PCR. The arrow indicates the duplication breakpoint product; affected individuals are marked with an asterisk. (C) Electropherogram of representative Sanger sequencing from an individual with ARCND showing the breakpoint nucleotide sequence. All figures are according to GRCh38 coordinates. ARCND, auriculocondylar syndrome.

Targeted resequencing of a 2.4 Mb region around *TWIST1* 31 revealed no potential pathogenic variants in *TWIST1*, but did detect a tandem duplication within *HDAC9* that was only present within the three affected family members tested (figure 1A and online supplemental figure 3). We designed primers to the sequences either side of the duplication and characterised the duplication breakpoint (figure 1B, C). The duplication spanned 430 302 bp (NC_000007.14:g.18437239_18867540dup) telomeric to *TWIST1*, covering most of the *HDAC9* gene. Multiple transcript isoforms of *HDAC9* are duplicated in their entirety (including transcript isoforms 3, 8, 9, 10 and 11); however, full-length catalytically active transcript isoforms^47^ (transcript isoforms 1, 5, 6 and 7) extend beyond the breakpoint and therefore are likely to be disrupted by this duplication. Breakpoint amplification and Sanger sequencing within the family demonstrated segregation of the duplication with the phenotype (figure 1B). All unaffected individuals were negative, suggesting full penetrance in this family (online supplemental figure 4).

Analysis of CNVs in DECIPHER (https://decipher.sanger.ac.uk/)^48^ revealed 19 patients with copy-number gains in the region. With the exception of a single small duplication (patient identification 276644), all the CNVs that overlap the one described here are much larger and encompass multiple genes. Nevertheless, 7 out of 19 have descriptions that include ear malformation among other features (patient identification: 393911, 393942, 395511, 396512, 280316, 396373 and 394346) include enhancer *eTW6* (Hs2307) that regulates the expression of *Twist1* 49 and other two regulatory elements (*eTW7* (Hs2306) and (*eTW5*) Hs644; VISTA Enhancer Browser). Similarly, 7 out of 19 DECIPHER patients presented with micrognathia (patient identification: 393911, 393942, 395511, 280316, 2363, 396373 and 394346); these duplications also include *eTW6*, except for individual 2363. Interestingly, one of the DECIPHER duplications (276644; 179 kb) is enclosed entirely within the ARCND duplicated region; however, this patient does not have ARCND clinical features (2021, Olivier Faivre, L., personal communication). We note that the non-overlapping sequence between patient 276644 and the ARCND duplication includes the aforementioned regulatory *TWIST1* elements (online supplemental figure 5 and table 6).

To explore how the duplication identified above, which is over 200 kb telomeric to the candidate gene *TWIST1*, could be pathogenic, we carried out a Capture-C analysis^50^ using the *TWIST1* promoter as the viewpoint. This demonstrated that there were multiple contacts between *TWIST1* and regions to either side. The highest frequency of interactions was telomeric of *TWIST1*, particularly within the *HDAC9* gene and the region spanned by the duplication (figure 1A). This implies that this region contains regulatory elements involved in the control of *TWIST1* expression, providing a possible pathogenic mechanism for the duplication.

### Analysis of the duplication in iPSC and craniofacial progenitor cells

To further investigate the pathogenicity of the duplication, we used an in vitro approach to model the disease, generating iPSC from affected and unaffected individuals in the family. The experimental design was based on recapitulating different stages of early embryonic development that are relevant to the ARCND phenotype, most particularly iNCC (iPSC-derived NCCs) and nMSC (iNCC-derived mesenchymal-cell like). All cell types were fully characterised and showed cellular specific expression of relevant markers and typical cell morphology (online supplemental figures 6–8).

### Expression analysis of *HDAC9*, *TWIST1* and ARCND-related markers

**Figure 2:**
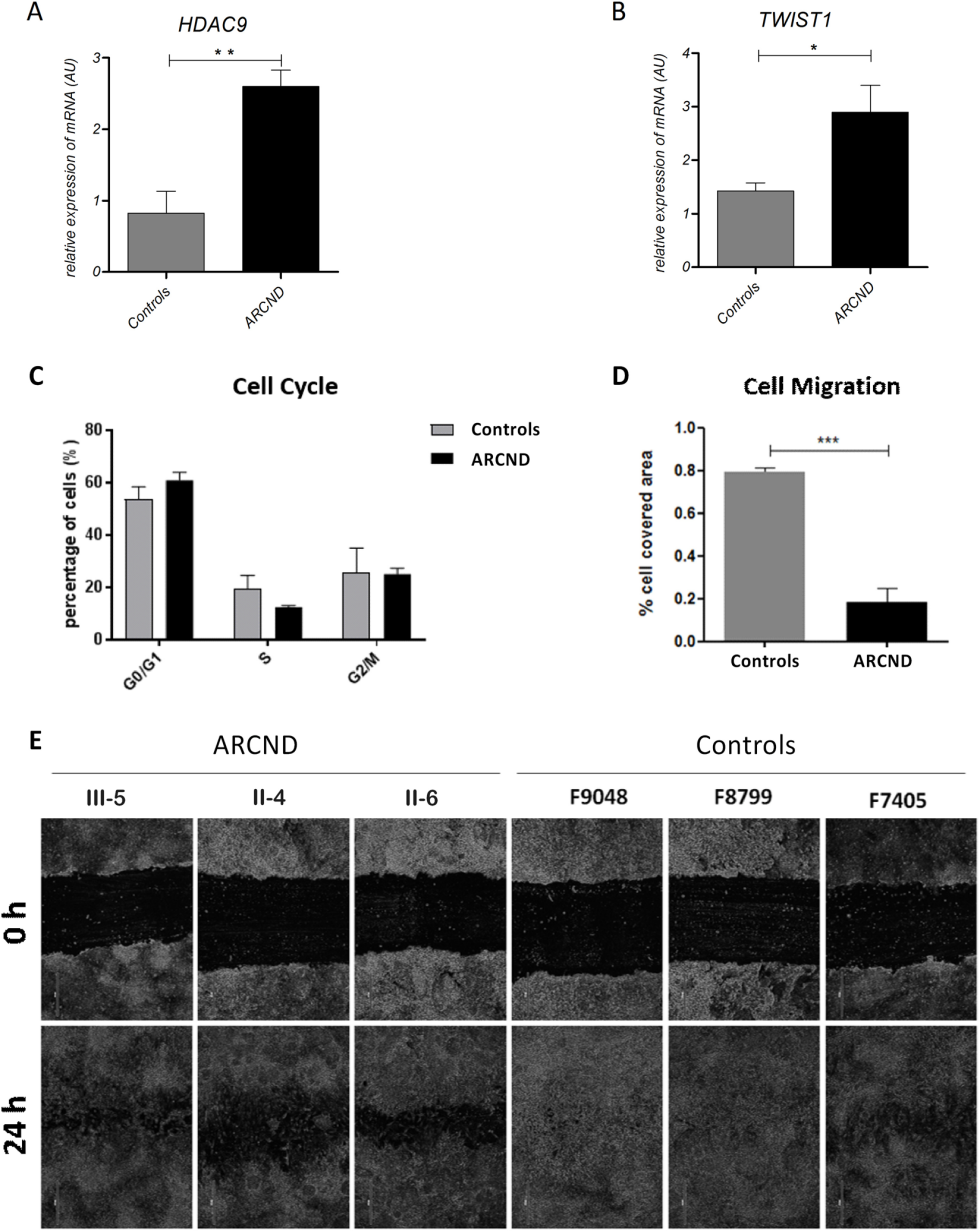
RT-QPCR assessment of (A) HDAC9 and (B) Twist1 showing upregulated expression in ARCND-derived NCC. Both genes showed statistically significant differences among controls and ARCND. **Two-tailed p=0.0094; *one-tailed p=0.0250, unpaired Student’s t-test. (C–E) Evaluation of cell cycle and cell migration in ARCND NCC. (C) Cell cycle assay, not statistically significant. (D) Bar graph depicting the rate of cell migration (cell-covered area, %) after 24 hours; data shown are representative of two independent assays and three independent measurements in each. ***Two-tailed, p=0.0009, Student’s t-test. (E) Representative phase-contrast micrographs acquired immediately after wounding at 0 and 24 hours. All values represent mean±SEM. ARCND, auriculocondylar syndrome; AU, arbitrary unit; NCC, neural crest cells.

Previous analysis of *HDAC9* has shown that it contains regulatory elements important for *TWIST1* expression.^49 51^ Together with our prioritisation and Capture-C analysis, this prompted us to investigate the expression of both of these genes in iPSC and derived cell types. *TWIST1* and *HDAC9* mRNA in iPSC did not show any difference between patients and controls (data not shown). However, an increase in *HDAC9* (3.15-fold, unpaired t-test p=0.009) and *TWIST1* (2.03-fold, unpaired t-test p=0.03) mRNA was observed in the ARCND-iNCC (figure 2A, B) compared with controls (unpaired t-test).

In order to evaluate if there is deregulation of the EDN1 pathway in the ARCND cells, we investigated the expression of the key downstream target of this pathway, *DLX5*,^15 52^ as well as other genes shown to be activated, *BARX1*, *NKX3.2*, *GSC*, *DLX3* and *HAND2*.^53 54^ Expression of *BARX1*, *NKX3.2* and *GSC* was not significantly different between controls and ARCND iNCCs (two-way ANOVA, p>0.05; online supplemental figure 9), while *HAND2*, *DLX3* and *DLX5* mRNA levels were too low to be measured (data not shown).

### Analysis of ARCND iNCC and nMSC function

To screen for cellular phenotypes, we assessed cell cycle and migration of ARCND iNCC compared with controls, as alterations in these cellular functions are considered to be underlying mechanisms in several NCC-related diseases.^17 55 56^ Although no significant difference in cell cycle distribution was detected between patient and control cells, a significant decrease in migratory capacity of ARCND iNCCs was observed compared with controls (4.3-fold decrease, Student’s t-test, p=0.0009) (figure 2C–E).

**Figure 3:**
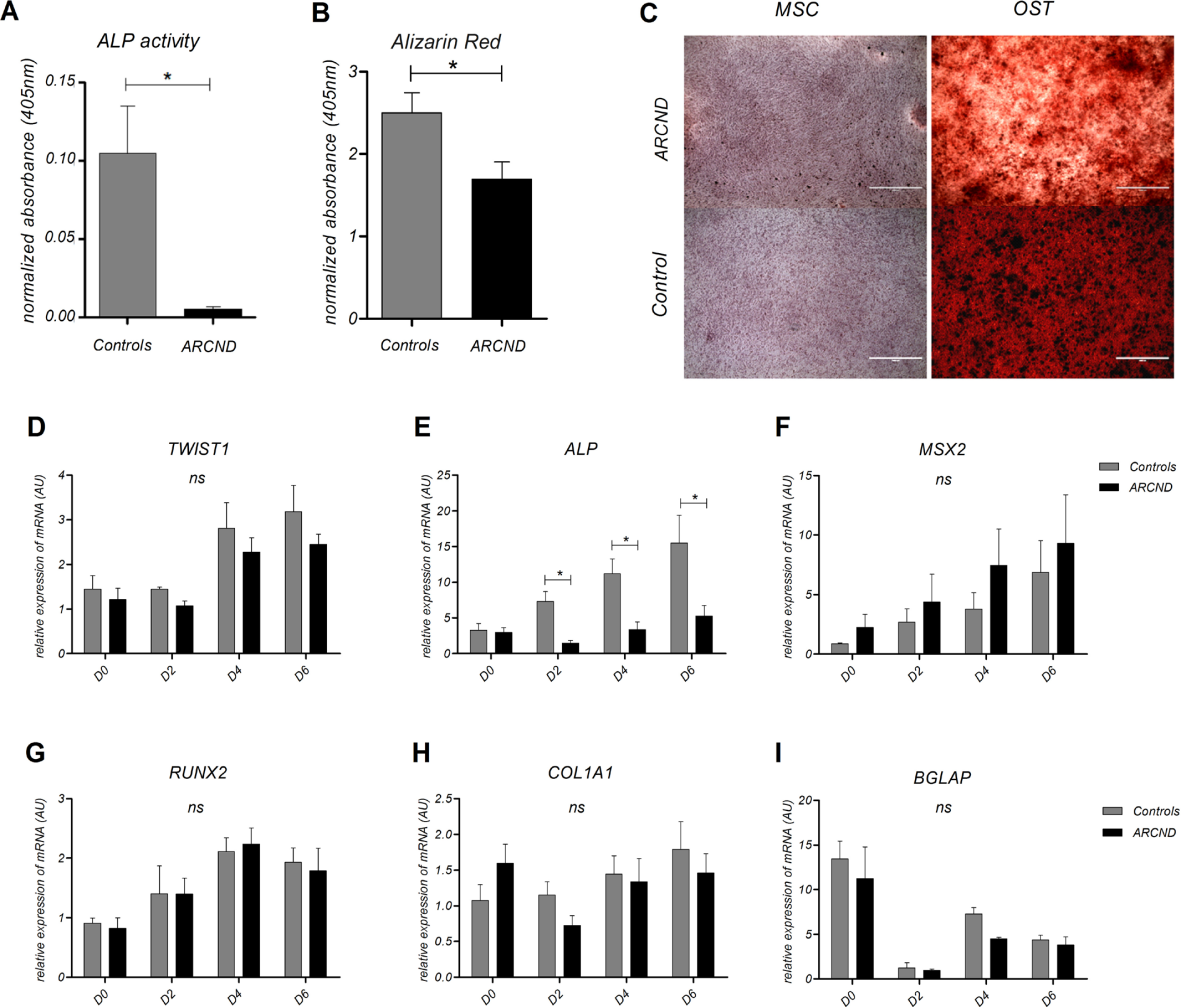
Evaluation of osteogenic potential in ARCND-mesenchymal stem cells (MSC). (A) Quantification of ALP enzymatic activity after 9 days and (B) alizarin red staining after 21 days of osteoinduction in ARCND-MSC in comparison with controls. Measurements from differentiated cells were normalised to paired, undifferentiated negative staining controls. (A and B) Student’s t-test, ALP activity two-sided and alizarin red one-sided (*p<0.05). (C) Representative alizarin red staining micrographs showing matrix mineralisation (in dark brown) of ARCND-MSC samples versus one representative control (osteogenic differentiation for 9 days); micrographs are shown paired to respective negative controls (undifferentiated cells). (D–I) Transcriptional profiles of *TWIST1* and osteogenic differentiation markers during the initial 6 days of osteoinduction. ALP was statistically significant (*p<0.05). *MSX2*, *RUNX2*, *COL1A1* and *BGLAP* did not show statistically significant differences (ns). All values represent mean±SEM. (D–I) Two-way ANOVA with Bonferroni post-tests. (C) Scale bars: 1000 µm. ALP, alkaline phosphatase; ANOVA, analysis of variance; ARCND, auriculocondylar syndrome; AU, arbitrary units; MSC, mesenchymal stem cells (undifferentiated cells); OST, osteogenic differentiation.

Marked mandibular hypoplasia is often seen in patients with ARCND, which could be caused by dysregulation of osteogenic differentiation. Therefore, we investigated this process in nMSCs. Our data showed that during osteogenic differentiation, ALP enzymatic activity was significantly diminished in ARCND-nMSCs after 9 days of osteoinduction (decrease of 20.3-fold, paired t-test p=0.029; figure 3A, B). In addition, alizarin red staining revealed a subtle decrease in matrix mineralisation in ARCND-nMSCs in comparison with controls (t-test p<0.05; figure 3C). Next, we assessed the expression of key osteogenesis genes (figure 3D–I). *ALP* showed a statistically significant downregulation (p=0.035), whereas *MSX2* expression was higher in ARCND-nMSC compared with the controls, although not reaching statistical significance. No significant difference was seen in the expression of *RUNX2, TWIST1*, *BGLAP* and *COL1A1*. Together, these results indicate a delay or impairment of osteogenic differentiation.

## Discussion

In this work, we used linkage and resequencing analysis to reveal a new ARCND locus on chromosome 7. We identified a novel 430 kb CNV that duplicated sequences beginning ~280 kb telomeric of *TWIST1*, the gene prioritised as the best candidate for the phenotype in the linkage region. The CNV, which segregated with the disease in a four-generation large Brazilian family, duplicated possible regulatory element sequences within *HDAC9*, which we demonstrated, through a chromosome conformation capture assay, make contact with the *TWIST1* promoter. This aligns with previous studies of this region that show that mouse *Twist1* regulatory regions can be found within both introns and exons of the *Hdac9* gene.^49 51 57^ Notably, a 23 kb deletion that included three of these regulatory element sequences, which may also be bound by craniofacial transcription factors Lmx1b and Tfap2, was associated with a reduction in *Twist1* expression.^51^ Furthermore, rearrangements that leave the gene intact but remove regions telomeric of *TWIST1* lead to Saethre-Chotzen syndrome (SCS),^58–61^ which is caused by *TWIST1* haploinsufficiency. SCS is characterised by craniosynostosis of the coronal sutures, which is not found in ARCND. Overall, these findings underline the importance of this region for *TWIST1* regulation and suggest that duplication of these and other regulatory elements could be associated with altered *TWIST1* expression during development. Importantly, transgenic enhancer assays have demonstrated in both mouse and zebrafish that some of these regulatory elements can drive *Twist1* pharyngeal expression. For example, the enhancer known as *eTw6* 51 or Hs2307 (Vista Enhancer Browser^62^) that overlaps with *Hdac9* exon 19 drives the expression of *Twist1* in mouse E11.5 pharyngeal arches,^49 51^ providing a mechanism whereby rearrangements at this genomic locus could lead to the pharyngeal arch-related developmental abnormalities found in ARCND. Further support for a link between increased *TWIST1* expression and the developmental anomalies found in our ARCND family comes from overlap with the clinical features described in cases with three copies of the 7p chromosomal region. For example, micrognathia or small mandibles have been found in patients with partial trisomy 7p.^63–66^ We note, however, that our patients did not have large, open fontanelles, the hallmark feature of trisomy 7p which has been linked to triple dosage of *TWIST1*,^67^ which may reflect having three copies of regulatory elements rather than three copies of the gene itself. Similarly, a significant number of DECIPHER duplications (~40%) at this locus were also associated with ARCND overlapping features, despite the variable phenotype and incomplete penetrance of ARCND.^9 10 13 68^ Interestingly, one of the duplications (276644) was entirely contained within the ARCND duplication, but this CNV did not span the known *Twist1* regulatory element^51^ and the case was not associated with any features of ARCND. Altogether, these observations reinforce that the 430 kb *HDAC9* duplication is pathogenic and that altered expression of *TWIST1* might contribute to the ARCND phenotype.

To investigate the pathogenicity of the CNV, we used an iPSC-based approach to screen for molecular and cellular alterations associated with ARCND in the family. As the affected craniofacial structures in ARCND arise from the neural crest, we generated and analysed NCC derived from patient and control iPSCs and found upregulation of both *HDAC9* and *TWIST1*. Only the shorter, catalytically inactive forms of *HDAC9* 47 could be transcribed from within the duplication to account for the upregulation of this gene, while the larger transcripts (the catalytic domain is encoded by multiple exons at the 3′ end of the gene) are predicted to be disrupted. HDAC9, a class II histone deacetylase enzyme, usually associated with transcriptional repression,^69^ has been linked to many types of cancer such as glioblastoma, breast cancer and oral squamous cell carcinoma, and chronic disorders such as diabetes and osteoporosis.^70–72^ There is no evidence of a role of *HDAC9* in craniofacial development and it is not expressed in mouse E11.5 pharyngeal arch,^49^ although a role in bone development is possible as *Hdac9* expression has been shown to increase osteoclastogenesis and regulate osteogenesis,^73 74^ and a contribution to the development of the ARCND phenotype cannot be excluded. In contrast, the relevance of *TWIST1* in craniofacial development has been highlighted by the human conditions SCS^75 76^ and Sweeney-Cox syndrome,^77^ which are caused by pathogenic variants in this gene, as well as by studies of *Twist1* mouse models.^43 46^ Moreover, Twist1 directly inhibits Runx2,^78 79^ the master regulator of osteogenic differentiation,[Bibr R78] as well as downstream targets of Runx2 like bone sialoprotein.^80^ Its overexpression leads to reduced ossification^78 81^ and conditional inactivation of *Twist1* has demonstrated an essential role in the survival of NCC in mandibular development, as well as in ossification of the mandible leading to mandibular hypoplasia, condylar process loss and altered middle ear.^43 46^ Of note, a comparable mandibular phenotype is also observed in a mouse model deleted for the enhancer that regulates pharyngeal arch expression of Hand2,^82^ a bHLH transcription factor that dimerises with Twist1 in mandible development.^83 84^ Together, this supports the contention that deregulation of TWIST1, as we have shown in iNCCs, could contribute to the ARCND phenotype.

As *Twist1* knockout in different mutant animal models leads to defective NCC migration,^85 86^ we performed in vitro scratch assays on iNCC derived from affected family members and controls and found significant reduction in ARCND-iNCCs. Reduced migration in early NCC stages has been found in RCPS, a craniofacial disorder also characterised by underdevelopment of mandibles and shown to be related to altered neural crest functions.^17^ The reduced migration found in ARCND-iNCC is in contrast to previous data showing that reduced migration is associated with loss of Twist1 in mice,^85 86^ as opposed to increased expression, as found here. An explanation for this might be that the iNCCs modelled in this study are in an earlier developmental stage and/or lack factors necessary to activate endothelin signalling, which are secreted by tissues within the pharyngeal arches in vivo.^87^ This may account for the low *DLX5/6* expression observed in our experiments. Although the iNCC derivation protocol used here is biased towards the cranial neural crest lineage,^88^ expression profiling assays will be necessary to further clarify the positional identity and developmental stage of iNCCs. Our observations suggest that upregulation or downregulation of *TWIST1* levels can lead to reduced migration depending on the developmental stage. The migration defect could potentially explain ARCND features through a reduction in NCC reaching the first and second pharyngeal arches, resulting in malformed derivatives such as mandible and external ear, a mechanism that also seems to be involved in RCPS.^17^ In regard to *HDAC9*, even though its overexpression has been associated with increased proliferation and migration in cancer cells, to date this gene has not been associated with neural crest proliferation/migration or specification of craniofacial elements.

Furthermore, our osteogenesis analysis showed that ARCND-nMCS have defects in their ability to form bone. We observed significantly decreased levels and activity of ALP in nMSC-derived from affected family members resulting in a decrease in matrix mineralisation, which may suggest a delay in the process of mineralisation. Interestingly, this is in opposition to the findings in nMSC of RCPS, which showed increased mineralisation.^17^ Even though reduced iNCC migration was observed in ARCND and RCPS, the underdeveloped mandible observed in patients with these disorders may depend on different molecular pathways. Notably, *TWIST1* expression in ARCND-nMSC did not show significant differences during the osteogenic differentiation as compared with control cells. Decreased ossification could potentially be related to the dynamics of TWIST1 dimerisation at a previous stage of the cellular differentiation, as these cells were differentiated from iNCCs with higher *TWIST1* levels in patients as compared with controls. Increased expression levels of *TWIST1* in iNCCs would lead to an alteration in the ratios of TWIST1 homodimers and heterodimers (with E-proteins such as TCF3, TCF4 and TCF12). Studies of cranial sutures suggest an antagonistic relationship with TWIST1 homodimers activating FGFR2 and osteogenic genes for ossification, while TWIST1 heterodimers, for example TWIST1-TCF3, promote mesenchymal expansion.^80 89^ We should also consider as an additional contributing factor to deregulated osteogenesis the role of TWIST1 in regulating osteogenesis by its direct interaction with RUNX2 in preosteoblasts.^78^ Interestingly, overexpression of Hand2, a partner of Twist1 in mandible differentiation and an inhibitor of Runx2, leads to delayed ossification, characterised by ALP low levels,^90^ which is comparable with our findings. We speculate that the reduced iNCC migration and the delayed ossification in nMSC differentiation could relate to altered expression of TWIST1 in early stages of NCC that would depend on the availability of the bHLH class partners or inhibitor of DNA-binding (ID) proteins.^91^ Further studies are necessary to test these hypotheses.

In summary, our data suggest that a unique 430 kb tandem duplication at the *HDAC9/TWIST1* locus is pathogenic, causing deregulation of *TWIST1* expression, which leads to the development of ARCND features through compromised neural crest migration and osteogenic differentiation, thus representing a novel mechanism to be investigated in the aetiology of ARCND.

## Data Availability

All data relevant to the study are included in the article or uploaded as supplementary information. Data availability statement All data needed to evaluate the conclusions in the paper are present in the paper and/or the Supplementary Materials. Additional data related to this paper may be requested from the authors.
