# Supplementary material for: New locus underlying auriculocondylar syndrome (ARCND): 430 kb duplication involving TWIST1 regulatory elements
Source: J Med Genet. 2021 Nov 8;59(9):895–905. doi: 10.1136/jmedgenet-2021-107825 (PMC9411924; doi:10.1136/jmedgenet-2021-107825)
Supplement: Supplementary data [file jmedgenet-2021-107825supp001.pdf]

JOURNAL OF MEDICAL GENETICS

A new locus underlying Auriculocondylar syndrome: 400kb duplication involving *TWIST1* regulatory elements.

Vanessa L. Romanelli Tavares[1*], Sofia Ligia Guimarães-Ramos[1*], Yan Zhou[2], Cibele Masotti[1,3], Suzana A.M. Ezquina[1,4], Danielle P. Moreira[1], Henk Buermans[5], Renato S. Freitas[6], Johan T. den Dunnen[5], Stephen R. F. Twigg[2**], Maria Rita Passos-Bueno[1**].

[1]Human Genome and Stem Cell Research Center, University of São Paulo, Institute of Bioscience, Department of Genetics and Evolutionary Biology.

[2]Clinical Genetics Group, MRC Weatherall Institute of Molecular Medicine, University of Oxford, John Radcliffe Hospital, Oxford, United Kingdom.

[3]Molecular Oncology Center, Hospital Sírio-Libanês, São Paulo, Brazil (current location of the author).

[4]Centre for Cancer Genetic Epidemiology, University of Cambridge, Cambridge, United Kingdom (current location of the author).

[5]Leiden Genome Technology Center, Leiden University Medical Center, Leiden, Nederland.

[6]Centro de Atendimento Integral ao Fissurado Labial Palatal (CAIF), Curitiba, PR, Brasil.

* These authors contributed equally to the study.

** Corresponding authors: Maria Rita Passos-Bueno 106 Matão Street, 05508-090, São Paulo – SP, Brazil, Email: passos@ib.usp.br and Stephen R. F. Twigg, Clinical Genetics Group, MRC Weatherall Institute of Molecular Medicine, University of Oxford, Oxford, United Kingdom, Email: stephen.twigg@imm.ox.ac.uk

Romanelli Tavares VL, *et al. J Med Genet* 2022; 59:895–905. doi: 10.1136/jmedgenet-2021-107825

SUPPLEMENTARY MATERIAL

Cell Characterization

Total DNA was extracted from iPSC cultures with the use of a NucleoSpin Tissue kit (Macherey-Nagel), following the supplier's instructions. For PCR reactions, primers targeting the OriP gene present in the backbone of the episomal vectors were used (F 5′-TTCCACGAGGGTAGTGAACC-3′ and R 5′-TCGGGGGTGTTAGAGACAAC-3′), according to recommendations provided elsewhere (Epi5 Episomal iPSC Reprogramming Kit, Life Technologies). Multiplex ligation-dependent probe amplification (MLPA) analysis was performed with subtelomeric kits (P036 and P070; MRC-Holland), to detect chromosomal imbalances, as previously described [1].

iPSC were fixed in 4% paraformaldehyde for 20 minutes at room temperature, followed by permeabilization with PBS 0.2% Triton X-100 for 30 minutes at 4°C. Blocking was carried out with PBS 5% BSA for 1 hour at room temperature, followed by incubation with primary antibodies (Supplementary Table S3) in blocking solution overnight at 4°C. After washing 3x with PBS, cells were incubated with secondary antibodies in blocking solution at 4°C for 1 hour, in the dark, followed by two PBS washes and counterstaining with DAPI solution (Life Technologies) for 2 minutes at room temperature. After a final PBS wash, cells were analysed with a fluorescent microscope (Axiovision; Zeiss).

To assess the immunophenotype of NCCs and MSCs, cells were detached with Accutase and TrypLE Express respectively, and washed twice with 2 volumes of blocking solution (4% BSA in PBS without Ca^{2+} and Mg^{2+}). Cells were incubated with the conjugated antibodies (Supplementary Table S3) in blocking solution in the dark for 1 hour at 4°C, washed twice with PBS, and fixed in 1% paraformaldehyde/PBS. Antibody concentrations followed the manufacturer's recommendations. A minimum of 5,000 events were acquired in a FACS Aria II flow cytometer (BD Biosciences) and analyzed on Flowing software (v2.5) and Guava Express PRO (Millipore).

Microscope imaging revealed iPSC, NCC, and MSC each exhibiting their typical cell morphology, suggesting a successful differentiation and no apparent difference between patients and controls with regard to cell morphology. After reprogramming iPSC, all the cells exhibited a typical pluripotent stem-cell morphology and positive staining for both

pluripotent-markers SSEA-4 and Oct3/4. All iPSCs showed no detectable signs of aneuploidy or genomic integration of the episomal vectors, thus being a suitable model for our study purposes (Supplementary Figure S6).

NCCs induced from iPSC populations were positively stained for NCC markers p75 and HNK1 with proportions of double-positive cells ranging between 74.6 and 95.8%, in both control and patients' cells. Furthermore, RT-qPCR assays showed upregulation of NCC markers *PAX3*, *SOX10*, *ZIC1* and *TFAP2A*, when compared with the originating iPSCs (Supplementary Figure S7).

MSCs also exhibited typical and homogeneous mesenchymal immunophenotype, with positive staining (>82%) for mesenchymal markers CD73, CD166 and CD90, and negative staining (<5%) for endothelial marker CD31 (Supplementary Figure S8). Also, MSCs could be differentiated into mesenchymal derivatives in vitro, such as osteoblasts, in agreement with the expected mesenchymal stem cell phenotype seen in previous works from our group [2–5].

References

1 Jehee FS, Takamori JT, Vasconcelos Medeiros PF, Pordeus ACB, Latini FRM, Bertola DR, Kim CA, Passos-Bueno MR. Using a combination of MLPA kits to detect chromosomal imbalances in patients with multiple congenital anomalies and mental retardation is a valuable choice for developing countries. *Eur J Med Genet* 2011;**54**:e425–32.

2 Yeh E, Atique R, Ishiy F a a, Fanganiello RD, Alonso N, Matushita H, da Rocha KM, Passos-Bueno MR. FGFR2 mutation confers a less drastic gain of function in mesenchymal stem cells than in fibroblasts. *Stem Cell Rev* 2012;**8**:685–95.

3 Griesi-Oliveira K, Sunaga DY, Alvizi L, Vadasz E, Passos-Bueno MR. Stem cells as a good tool to investigate dysregulated biological systems in autism spectrum disorders. *Autism Res* 2013;**6**:354–61.

4 Kobayashi GS, Alvizi L, Sunaga DY, Francis-West P, Kuta A, Almada BVP, Ferreira SG, de Andrade-Lima LC, Bueno DF, Raposo-Amaral CE, Menck CF, Passos-Bueno MR. Susceptibility to DNA Damage as a Molecular Mechanism for Non-Syndromic Cleft Lip and Palate. *PLoS One* 2013;**8**:e65677.

5 Miller EE, Kobayashi GS, Musso CM, Allen M, Ishiy FAA, de Caires LC, Goulart E, Griesi-Oliveira K, Zechi-Ceide RM, Richieri-Costa A, Bertola DR, Passos-Bueno MR, Silver DL. EIF4A3 deficient human iPSCs and mouse models demonstrate neural crest defects that underlie Richieri-Costa-Pereira syndrome. *Hum Mol Genet* 2017;**26**:2177–91.

Supplementary Table S1: Summary of clinical features of Family F1 diagnosed with ARCND and previously reported in Masotti *et al*., 2008.

	Family F1
**Original description**	Masotti *et al* ., 2008, referred to F1
**Diagnosis / Inheritence**	ARCND / AD
**Familial recurrence**	+
**Consanguinity**	-
**Gender of affected patients**	F (8/11); M (3/11)
Neuropsychomotor development delay	0/10
Apnea	4/10
Respiratory difficulties	4/10
**FACE**	
Facial asymmetry	6/10
Prominent cheeks	8/10
**MOUTH**	
Microstomia	8/10
Abnormal palate; cleft palate	3/10 ; 1/10
Excess soft tissue / Atypical uvula	NE
Glossoptosis	4/9
**MANDIBLE**	
Micrognathia	6/10
**CONDYLE**	
Mandibular condyle abnormality	3/4
**EARS**	
Other auricular dysplasia	2/10
QME	8/10
Post-auricular tags	2/10
Hearing loss	6/6

F - female; M - male; ARCND - auriculocondylar syndrome; AD - autosomic dominant;
NE - not evaluated.

Romanelli Tavares VL, *et al. J Med Genet* 2022; 59:895–905. doi: 10.1136/jmedgenet-2021-107825

Supplementary Table S2. Training lists used to run ENDEAVOUR prioritization genes. (A) Genes that have an important role in formation of the structures mainly affected in ARCND patients - ears, mandible, and mandibular condyle. (B) Genes involved in the embryonic developmental processes of the structures affected in ARCND patients; including those important in neural crest development and formation of the first and second pharyngeal arches.

Training list A	Training list B	
DLX3	*BAPX1*	*NRP1*
DLX4	*DLX3*	*NRP2*
DLX5	*DLX5*	*OTX2*
DLX6	*DLX6*	*PAX3*
EDN1	*EDN1*	*PAX7*
EYA1	*FGF8*	*PITX1*
FGF8	*GSC*	*RHOB*
GSC	*HAND2*	*SNAI1*
HAND2	*LHX6*	*SOX9*
OTX2		
RUNX2		

Romanelli Tavares VL, *et al. J Med Genet* 2022; 59:895–905. doi: 10.1136/jmedgenet-2021-107825

Supplementary Table S3: Antibodies used for iPSC, NCC, and MSC characterization, through immunofluorescence and/or flow cytometry.

Cell type	Technique	Antibody	Company (catalog#)
iPSC	immunofluorescence	primary Anti-OCT4 antibody	Abcam (ab19857)
iPSC	immunofluorescence	primary Anti-SSEA4 antibody	Abcam (ab16287)
iPSC	immunofluorescence	secondary Goat anti-Rabbit IgG (H+L) antibody Alexa Fluor 546 conjugate	Life Technologies(A-11010)
iPSC	immunofluorescence	secondary Goat anti-Mouse IgG (H+L) Antibody, Alexa Fluor 488 conjugate	Life Technologies(A-11001)
NCC	flow citometry	IgM k FITC Mouse Anti-Human CD57	BD Pharmingen (561906)
NCC	flow citometry	IgG1 k Alexa Fluor 647 Mouse Anti-Human CD271	BD Pharmingen (560877)
NCC	flow citometry	FITC Mouse IgM k isotype control	BD Pharmingen (555583)
NCC	flow citometry	Alexa Fluor 647 Mouse IgG1 k isotype control	BD Pharmingen (557714)
MSC	flow citometry	FITC Mouse Anti- Human CD31	BD Pharmingen (555445)
MSC	flow citometry	APC Mouse Anti-Human CD73	BD Pharmingen (560847)
MSC	flow citometry	PE Mouse Anti-Human CD90	BD Pharmingen (555596)
MSC	flow citometry	FITC Mouse Anti-Human CD105	BD Pharmingen (551443)
MSC	flow citometry	PE Mouse Anti-Human CD166	BD Pharmingen (559263)
MSC	flow citometry	FITC Mouse IgG1 κ Isotype Controls	BD Pharmingen (555748)
MSC	flow citometry	PE Mouse IgG1 κ Isotype Control	BD Pharmingen (554681)
MSC	flow citometry	APC Mouse IgG1 κ Isotype Controls	BD Pharmingen (555749)

Romanelli Tavares VL, *et al. J Med Genet* 2022; 59:895–905. doi: 10.1136/jmedgenet-2021-107825

Supplementary Table S4. Real time PCR primer sequences.

Gene	Forward sequence 5′-3′	Reverse sequence 5′-3′
ALP	GATACAAGCACTCCCACTTCATCTG	CTGTTCAGCTCGTACTGCATGTC
BARX1	GAAGTGGAAGAAAATAGTGCTGC	CGCTCGTTGGAATTGAGTTCT
BGLAP	GGCGCTACCTGTATCAATGG	TCAGCCAACTCGTCACAGTC
COL1A1	TGCACCACCAACTGCTTAGC	GGCATGGACTGTGGTCATGA
DLX3	CCGCGTACGATCTACTCCAG	ATTTTCACCTGTGTCTGCGTG
DLX5	ACCAGCCAGAGAAAGAAGTGAC	CCTTCTCTGTAATGCGGCCA
GAPDH	ATCACCATCTTCCAGGAGCG	GGGCAGAGATGATGACCCTTT
GSC	CGAGGAGAAAGTGGAGGTCTG	AGCTGTCCGAGTCCAAATCG
HAND2	CACCAGCTACATCGCCTACC	GATTTCGTTCAGCTCCTTCTTCC
*HDAC9**	GCATGAGAACTTGACACGG	TTGTTGCTGTTTTATGGCTAGAAG
HMBS	AGCTTGCTCGCATACAGACG	AGCTCCTTGGTAAACAGGCTT
MSX2	GAAGACGGAGCACCGTGGATA	TCCAAGGCTAGAAGCTGGGATG
NKX3.2	ACCCTTAAACAGGTGATCCCAC	GTGCCATTAAGGAGGCGAAAAG
RUNX2	AGTGGACGAGGCAAGAGTTTC	GTTCCCGAGGTCCATCTACTG
TBP	GTGACCCAGCATCACTGTTTC	GCAAACCAGAAACCCTTGCG
TWIST1	GACCTAGATGTCATTGTTTCCAGA	GCCTGTCTCGCTTTCTCTTTTA

**HDAC9* primers were designed to exons covered by duplication

Supplementary Table S5. Gene prioritization using the ENDEAVOUR program. *TWIST1* was the top ranked gene, with a p-value equal to 0.000527 and 0.000273, using training gene lists A and B, respectively. The table show the first twenty genes.

Rank position	Results using training list A			Results using training list B		
	Gene Symbol	Gene main identifier	P-value	Gene Symbol	Gene main identifier	P-value
1	*TWIST1*	ENSG00000122691	5.273E-04	*TWIST1*	ENSG00000122691	2.730E-04
2	*EVX1*	ENSG00000106038	1.098E-03	*MEOX2*	ENSG00000106511	4.619E-04
3	*HOXA1*	ENSG00000105991	1.918E-03	*SP4*	ENSG00000105866	2.092E-03
4	*SP4*	ENSG00000105866	2.025E-03	*EVX1*	ENSG00000106038	2.565E-03
5	*HOXA3*	ENSG00000105997	2.478E-03	*HOXA2*	ENSG00000105996	3.720E-03
6	*HOXA13*	ENSG00000106031	3.431E-03	*HOXA1*	ENSG00000105991	4.157E-03
7	*MEOX2*	ENSG00000106511	3.701E-03	*HOXA13*	ENSG00000106031	4.340E-03
8	*HOXA11*	ENSG00000005073	4.426E-03	*HOXA3*	ENSG00000105997	6.163E-03
9	*ETV1*	ENSG00000006468	4.462E-03	*HOXA11*	ENSG00000005073	6.485E-03
10	*HOXA2*	ENSG00000105996	5.953E-03	*HOXA7*	ENSG00000122592	8.270E-03
11	*HOXA7*	ENSG00000122592	6.496E-03	*NPY*	ENSG00000122585	1.268E-02
12	*HOXA5*	ENSG00000106004	1.776E-02	*HOXA9*	ENSG00000078399	1.757E-02
13	*ITGB8*	ENSG00000105855	1.807E-02	*HOXA5*	ENSG00000106004	2.039E-02
14	*HOXA9*	ENSG00000078399	2.059E-02	*ETV1*	ENSG00000006468	2.143E-02
15	*NPY*	ENSG00000122585	2.539E-02	*GPNMB*	ENSG00000136235	2.587E-02
16	*SOSTDC1*	ENSG00000171243	2.823E-02	*HOXA4*	ENSG00000197576	2.818E-02
17	*IGF2BP3*	ENSG00000136231	3.052E-02	*AGR2*	ENSG00000106541	3.051E-02
18	*AGR2*	ENSG00000106541	3.225E-02	*SOSTDC1*	ENSG00000171243	3.120E-02
19	*GPNMB*	ENSG00000136235	3.493E-02	*ITGB8*	ENSG00000105855	3.203E-02

Romanelli Tavares VL, *et al. J Med Genet* 2022; 59:895–905. doi: 10.1136/jmedgenet-2021-107825

Supplementary Table S6. Patients from the DECIPHER database with copy-number gains that overlap the duplicated region found in the present ARCND family. ND − not described. (*) patient with a duplication within the ARCND duplicated region. Italic and underlined – non-specific auricular malformation. Bold – micrognathia, an alteration also found in ARCND. Refer to the main text and Supplementary Figure S5 for details. All data were extracted from DECIPHER database (https://decipher.sanger.ac.uk/ ; Firth et al. 2009).

	DECIPHER ANNOTATIONS						
**Patient ID**	**Phenotype**	**CRCh37/hg19**	**CRCh38/hg38**	**Size**	**Gene**	**Inheritance/Genotype**	**Pathogenicity/Contribution**
**393911**	Brachycephaly, Camptodactyly of finger, Downslanted palpebral fissures, Downturned corners of mouth, Epicanthus, Hypertelorism, Intellectual disability, Limitation of knee mobility, *Low-set ears*, **Micrognathia,** Muscular hypotonia, *Posteriorly rotated ears*, Prominent occiput, Rocker bottom foot, Short nose, Wide intermamillary distance, Wide nasal bridge	7:10239-20826760	7:10239-20787141	20.78 Mb	182	Imbalance arising from a balanced parental rearrangement Heterozygous	Likely pathogenic
**393942**	*Abnormality of the pinna*, Brachycephaly, Camptodactyly of finger, Convex nasal ridge, *Crumpled ear*, Deep philtrum, Delayed closure of the anterior fontanelle, Downslanted palpebral fissures, Epicanthus, Finger clinodactyly, Frontal bossing, High palate, Hypertelorism, *Low-set ears*, Macroglossia, **Micrognathia,** Microphthalmia, Muscular hypotonia, Narrow mouth, Prominent glabella, Rocker bottom foot, Short neck, Short nose, Single transverse palmar crease, Sparse hair, Wide cranial sutures, Wide intermamillary distance, Wide nasal bridge	7:10239-20826760	7:10239-20787141	20.78 Mb	182	Imbalance arising from a balanced parental rearrangement Heterozygous	Likely pathogenic
**395511**	*Abnormality of the outer ear*, Anteverted nares, Cat cry, Cryptorchidism, Depressed nasal bridge, Finger clinodactyly, **Micrognathia,** Micropenis, Muscular hypotonia, Patent ductus arteriosus, *Prominent antihelix*, Prominent occiput, Ptosis, Rocker bottom foot, Short neck, Short nose, Short stature, Single transverse palmar crease, Sloping forehead, Thin lower lip vermilion, Thin upper lip vermilion, Wide nasal bridge	7:10239-20826760	7:10239-20787141	20.78 Mb	182	Imbalance arising from a balanced parental rearrangement Heterozygous	Likely pathogenic
**396512**	*Abnormality of the pinna*, Brachycephaly, Broad palm, Delayed closure of the anterior fontanelle, Delayed speech and language development, Depressed nasal bridge, Downslanted palpebral fissures, Downturned corners of mouth, Furrowed tongue, Genu valgum, Intellectual disability, *Low-set ears*, Malar flattening, Muscular hypotonia, Proximal placement of thumb, Talipes equinovalgus	7:10239-20826760	7:10239-20787141	20.78 Mb	182	Imbalance arising from a balanced parental rearrangement Heterozygous	Likely pathogenic
**280316**	Absent speech, Constipation, Global developmental delay, Intellectual disability, severe, Long fingers, *Low-set ears*, **Micrognathia,** Muscular hypotonia, **Narrow mouth,** Pancreatitis, Thoracolumbar scoliosis	7:10239-25112979	7:10239-25073360	25.06 Mb	230	Unknown Heterozygous	ND
**2363**	Anteverted nares, Arachnoid cyst, Delayed cranial suture closure, Feeding difficulties in infancy, Hypertelorism, Hypoplasia of the corpus callosum, Intellectual disability, **Micrognathia,** Plagiocephaly, Wide nasal bridge	7:54215-18545043	7:54215-18505420	18.45 Mb	167	Unknown Heterozygous	ND
**259691**	Intellectual disability	7:2996438-26613851	7:2956804-26574232	23.62 MB	194	De novo heterozygous	ND
**250623**	ND	7:9080957-29334149	7:9041327-29294533	20.25	161	Unknown Heterozygous	ND

… (continuation of the Supplementary Table S6)

DECIPHER ANNOTATIONS							
**Patient ID**	**Phenotype**	**CRCh37/hg19**	**CRCh38/hg38**	**Size**	**Gene**	**Inheritance/Genotype**	**Pathogenicity/Contribution**
**383307**	Abnormal facial shape, Intellectual disability, mild, Iris coloboma, Retinal coloboma, Right hemiplegia, Seizure, Short stature	7:10341312-22824687	7:10301685-22785068	12.48	74	De novo heterozygous	Pathogenic full
**366699**	Failure to thrive, Intellectual disability, mild, Microcephaly	7:10795079-19802691	7:10755452-19763068	9.01 Mb	49	Patternally inherited Heterozygous	Likely benign Uncertain
**396373**	Abnormal immunoglobulin level, Abnormality of cardiovascular system morphology, *Abnormality of the pinna , Aplasia/Hypoplasia of the earlobes ,* Camptodactyly of finger, Deep palmar crease, Delayed closure of the anterior fontanelle, Delayed speech and language development, Downslanted palpebral fissures, Hip dislocation, Intellectual disability, Long philtrum, Microcephaly, **Micrognathia,** Muscular hypotonia, *Prominent antihelix, Prominent ear helix ,* Proportionate short stature, Small for gestational age, Telecanthus, Tricuspid regurgitation	7:13326761-30526760	7:13287136-30487144	17.20 Mb	162	De novo heterozygous	Likely pathogenic
**280487**	Global developmental delay	7:14775680-24813558	7:14736055-24773939	10.04 Mb	85	De novo Heterozygous	ND
**326652**	Intellectual disability, Macrocephaly	7:15614415-24597968	7:15574790-24558349	8.98 Mb	79	Paternally inherited Heterozygous	Uncertain Uncertain
**300578**	Atrial septal defect, Neonatal hypotonia	7:15614415-24621160	7:15574790-24581541	9.01 Mb	80	Unknown Heterozygous	Pathogenic full
**394346**	Abnormal thumb morphology, Abnormality of finger, Downslanted palpebral fissures, Genu valgum, High palate, Intellectual disability, *Low-set ears ,* Macrotia, **Micrognathia,** Micropenis, Muscular hypotonia, Premature birth, *Protruding ear ,* Ptosis	7:16426760-37226760	7:16387135-37187155	20.80 Mb	214	Unknown Heterozygous	Likely pathogenic
**288069**	Intellectual disability	7:17299010-18730173	7:17259386-18690550	1.43 Mb	6	Unknown Heterozygous	Uncertain
**288080**	Intellectual disability, Microcephaly	7:17299010-18730173	7:17259386-18690550	1.43 Mb	6	Maternally inherited Heterozygous	Uncertain
**255320**	Abnormal facial shape, **Cleft palate**, Cognitive impairment	7:17795419-20010339	7:17755795-19970716	2.21 Mb	13	Inherited from parent with similar phenotype to child Heterozygous	ND
***276644**	ND (however, there is no ear or mandible malformation – personal communication from Laurence Olivier-Faivre)	7:18520874-18700051	7:18481251-18660428	179.18 Kb	*HDAC9*	Unknown heterozygous	ND

Reference: Firth HV, Richard SM, Bevan AP, et al (2009) DECIPHER: Database of Chromosomal Imbalance and Phenotype in Humans using Ensembl Resources. Am.J.Hum.Genet 84 524-533 (http://doi.org/10/1016/j.ajhg.2009.03.010)

Romanelli Tavares VL, *et al. J Med Genet* 2022; 59:895–905. doi: 10.1136/jmedgenet-2021-107825

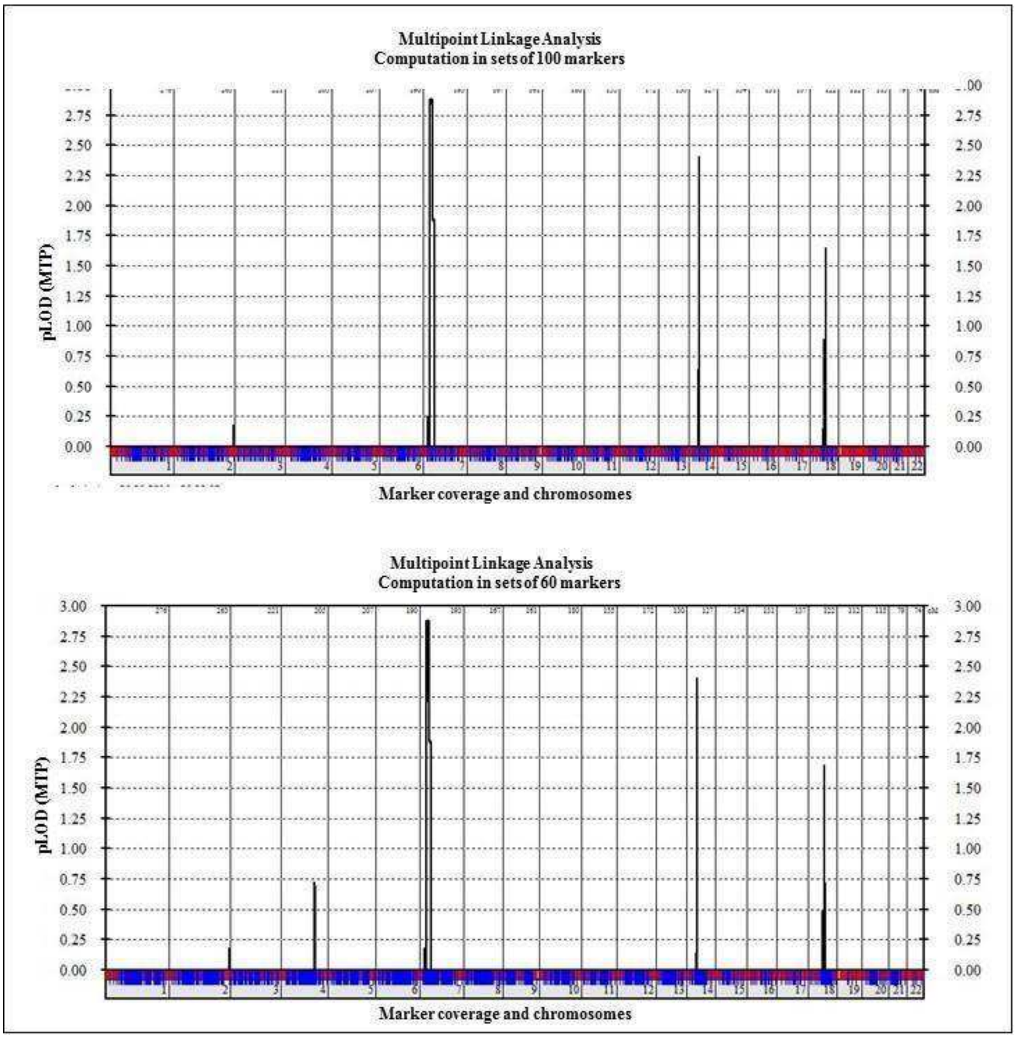

Supplementary Figure S1. Multipoint linkage analysis performed with GeneHunter using sets of 100 and 60 markers, shown respectively at the top and bottom of the figure. The start/end of each marker set is shown through the blue bars along the x-axis. The highest LOD score was found on Chromosome 7 (see text for details).

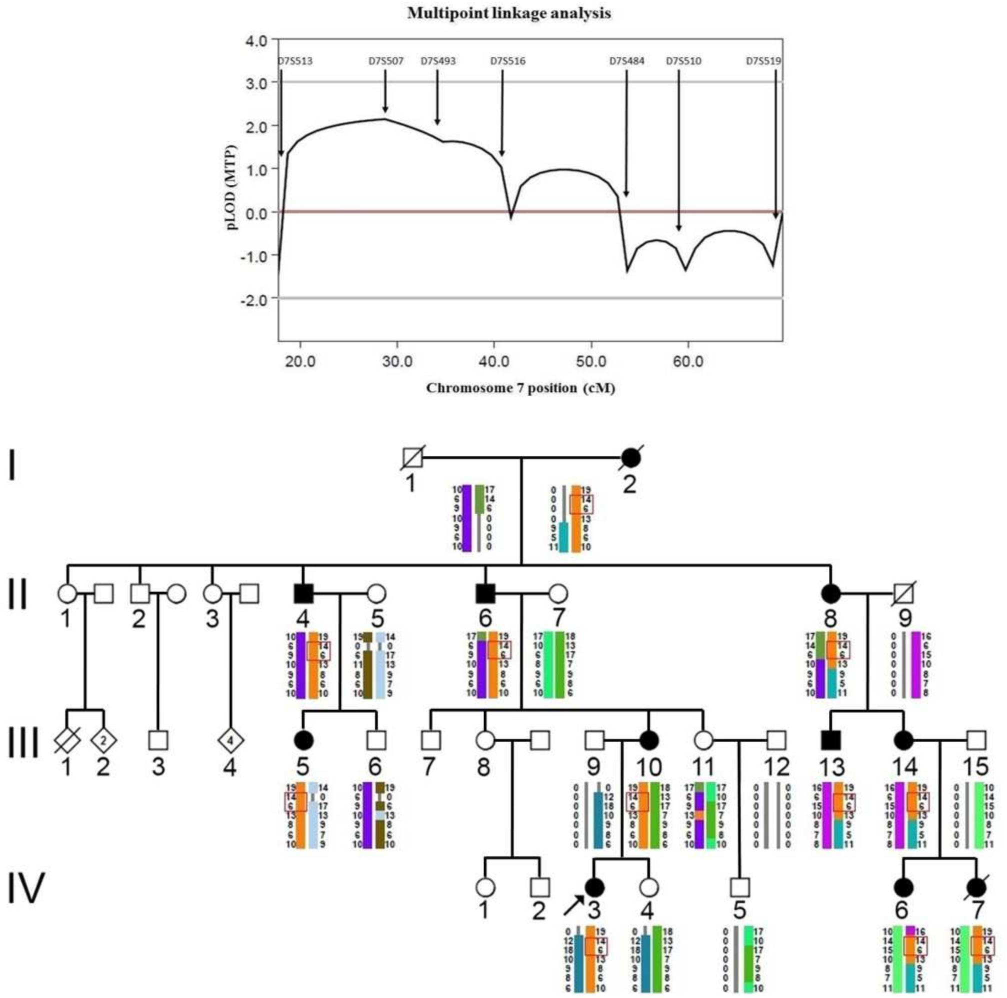

Supplementary Figure S2. Microsatellite markers genotyping. **Top**, multipoint linkage analysis performed with Merlin using microsatellites genotypes. D7S513 (17.7 cM), D7S507 (28.7 cM), D7S493 (34.7 cM), D7S516 (41.7 cM), D7S484 (53.5 cM), D7S510 (59.9 cM), and D7S519 (69.0 cM). LOD score positive region from D7S513 to D7S484 at chr7:11651237-35284906 (hg19). **Bottom**, haplotypes, constructed using HaploPainter, showing segregation of microsatellites markers in the candidate region among affected patients (D7S513 to D7S516). Haplotypes for individuals I-1, I-2, II-6, III-4, III-6, III-9 e IV-5 were inferred by Merlin software once their DNA samples were unavailable. This analysis narrowed down linkage region from chr7:14395902-32017194 to chr7:14395902-28158440 (hg38).

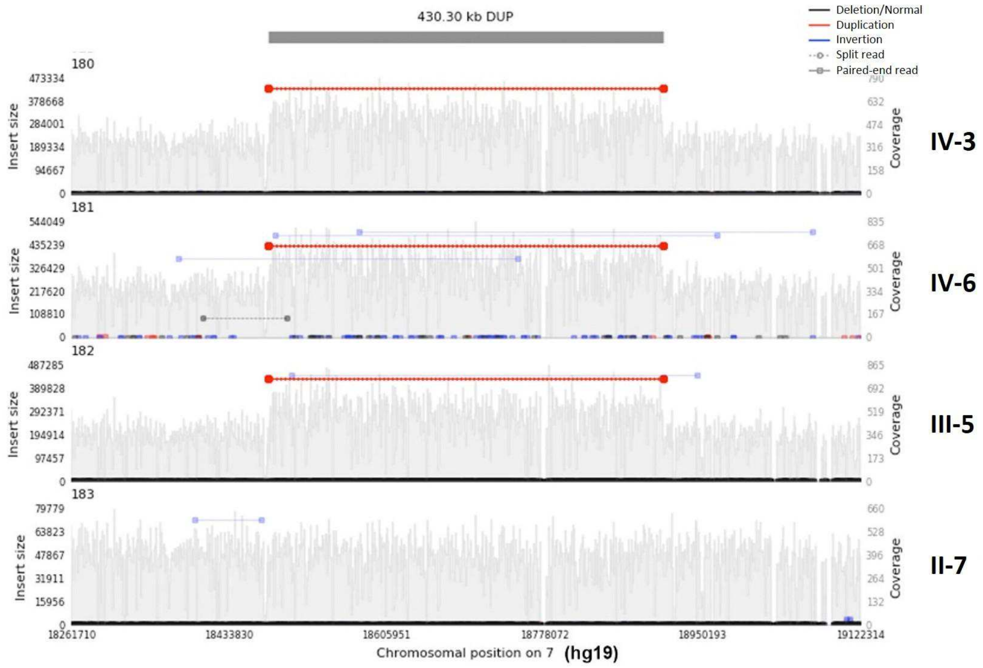

Supplementary Figure S3 - Targeted resequencing of a 2.4 Mb region around *TWIST1*. A *tandem* duplication of 430.30 kb within *HDAC9* is seen in affected patients IV-3, IV-6, and III-5. Individual II-7 is unaffected and does not have the duplication. Image captured with Samplot (https://www.biorxiv.org/content/10.1101/2020.09.23.310110v3).

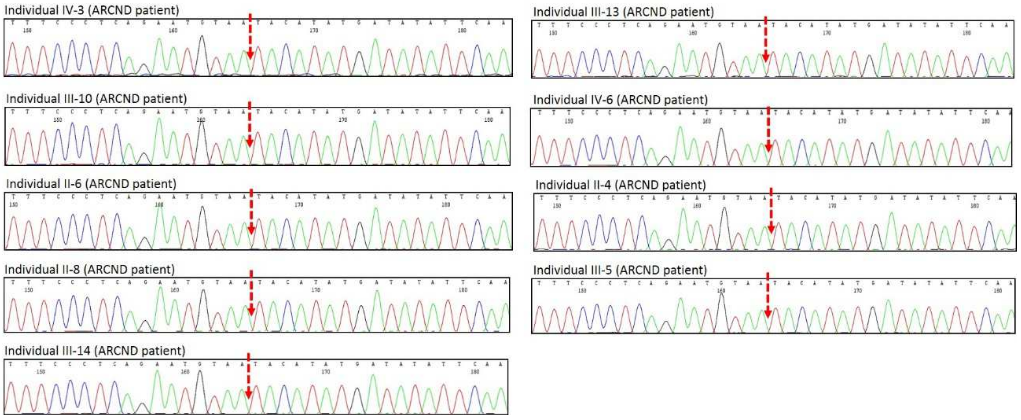

Supplementary Figure S4. *HDAC9* breakpoint evaluation in patients of F1 family. Sanger sequencing of ARCND patients showing the presence of the breakpoint region, in affected individuals only. The exact breakpoint was mapped using UCSC Blat.

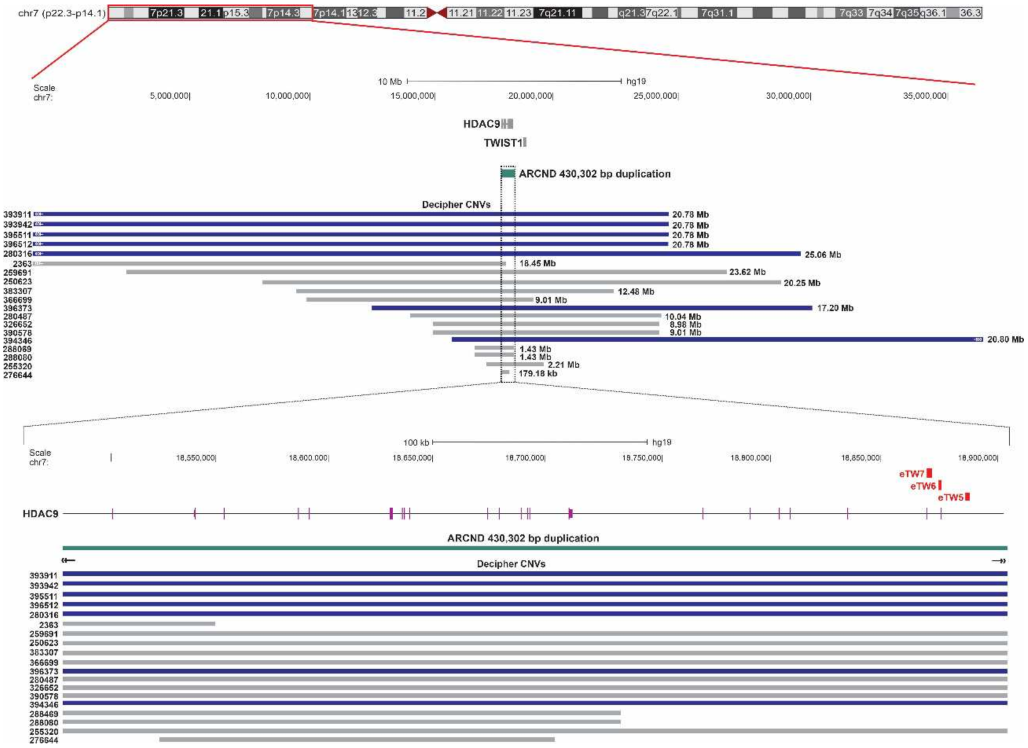

Supplementary Figure S5. UCSC screenshot scheme of chr7:10,239-37,226,760 (hg19; **above**) showing the surrounding region of ARCND 430 kb and DECIPHER database CNVs. **Bellow**, there is a zoom in the ARCND 430 Kb duplication region (located at chr7:18,476,861-18,907,163 hg19), along with the *HDAC9* scheme. We can see that not all isoforms would be disrupted by the ARCND duplication (refer to the main text). DECIPHER track was filtered by variant class to only display copy-number gain, duplication, and duplication/trip. After that, only duplications that overlap with the ARCND 430 kb CNV were included. Blue CNVs bars represent the ones with some type of auricular malformation described in the DECIPHER database. DECIPHER patients without described ear malformation are depicted as gray bars (CNVs). Refer to the main text and Supplementary Table S5 for details.

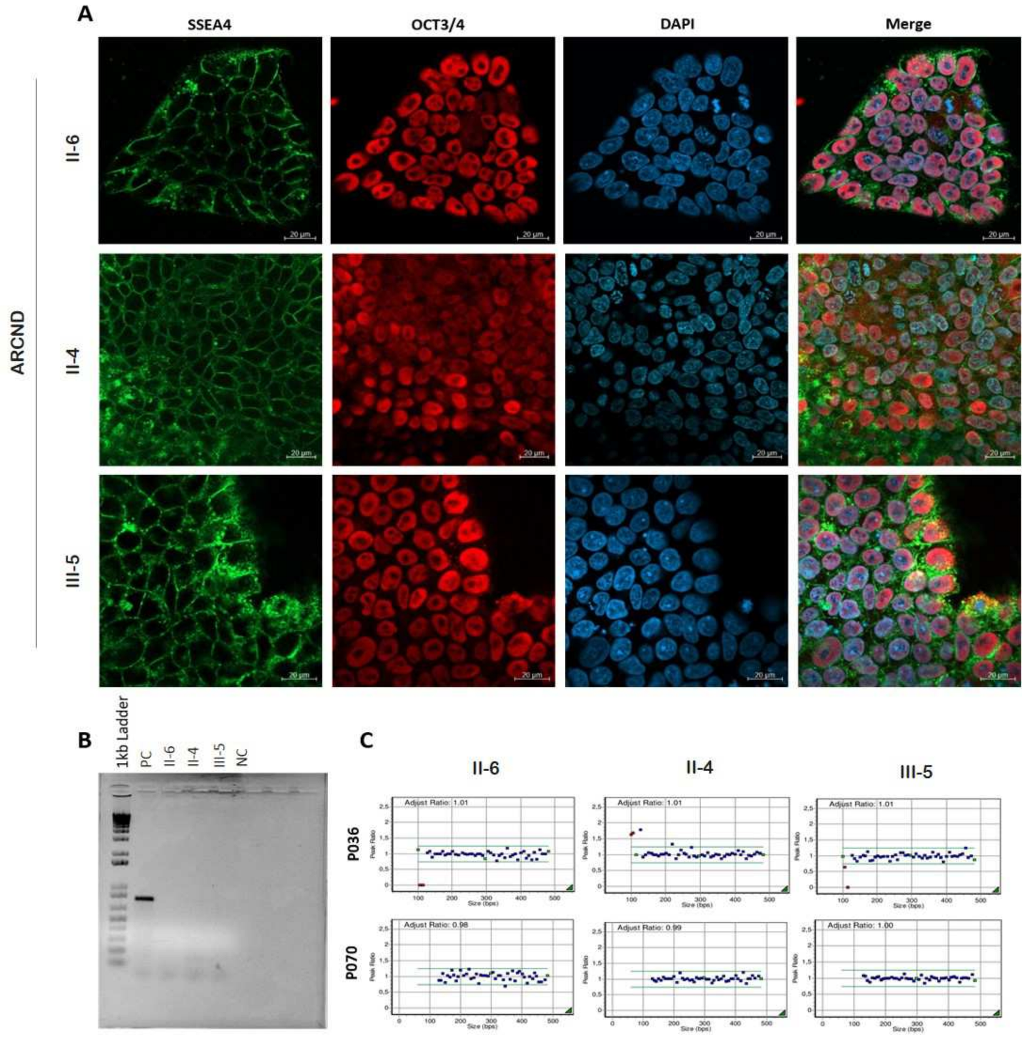

Supplementary Figure S6. Characterization of Induced Pluripotent Stem Cells (iPSC) reprogrammed from ARCND erythroblasts cells. **(A)** Immunofluorescence staining of pluripotency markers OCT3/4 (red) and SSEA-4 (green) in iPSCs (10x magnification); DAPI nuclear staining is in blue. **(B)** End-point PCR for vector backbone gene *OriP;* DNA from one iPSC line in which genomic integration had been detected was used as positive control (PC); (NC) negative control. **(C)** MLPA analysis with peak ratios for subtelomeric probes (blue dots) and control probes (green dots) using P036 and P070 kits showing no evidence of alterations.

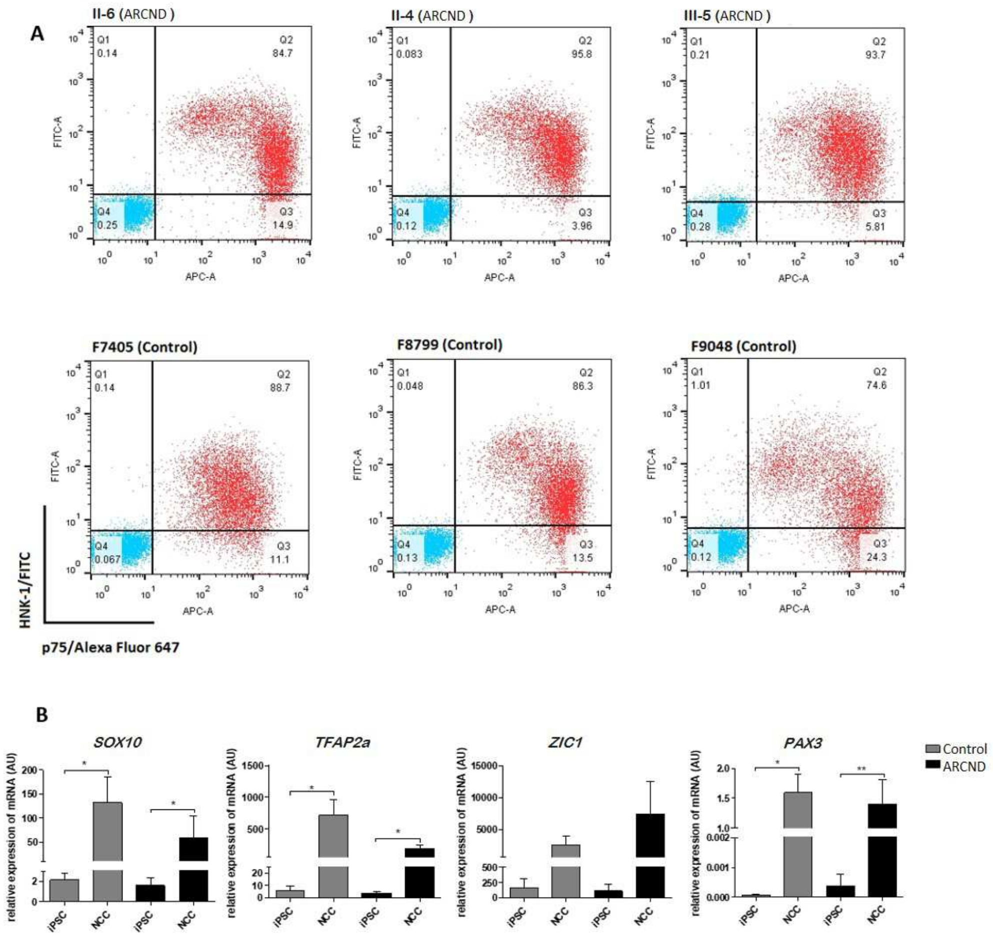

Supplementary Figure S7. Characterization of iPSC-derived neural crest cells. (**A**) Biparametric flow cytometry dot plots for HNK-1/FITC and p75/Alexa Fluor 647 expression in ARCND and control cells. Values in upper right quadrants represent p75+/HNK-1+ events. (**B**) RT-qPCR assessment of neural crest markers (*PAX3*, *SOX10*, *ZIC1* and *TFAP2A*) and NCCs from controls(gray) and ARCND patients (black) with n=number of biological samples; values represent mean ± SEM, (**) *p*-value< 0.01, (*) *p*-value < 0.05, Student’s t-test.

Romanelli Tavares VL, *et al. J Med Genet* 2022; 59:895–905. doi: 10.1136/jmedgenet-2021-107825

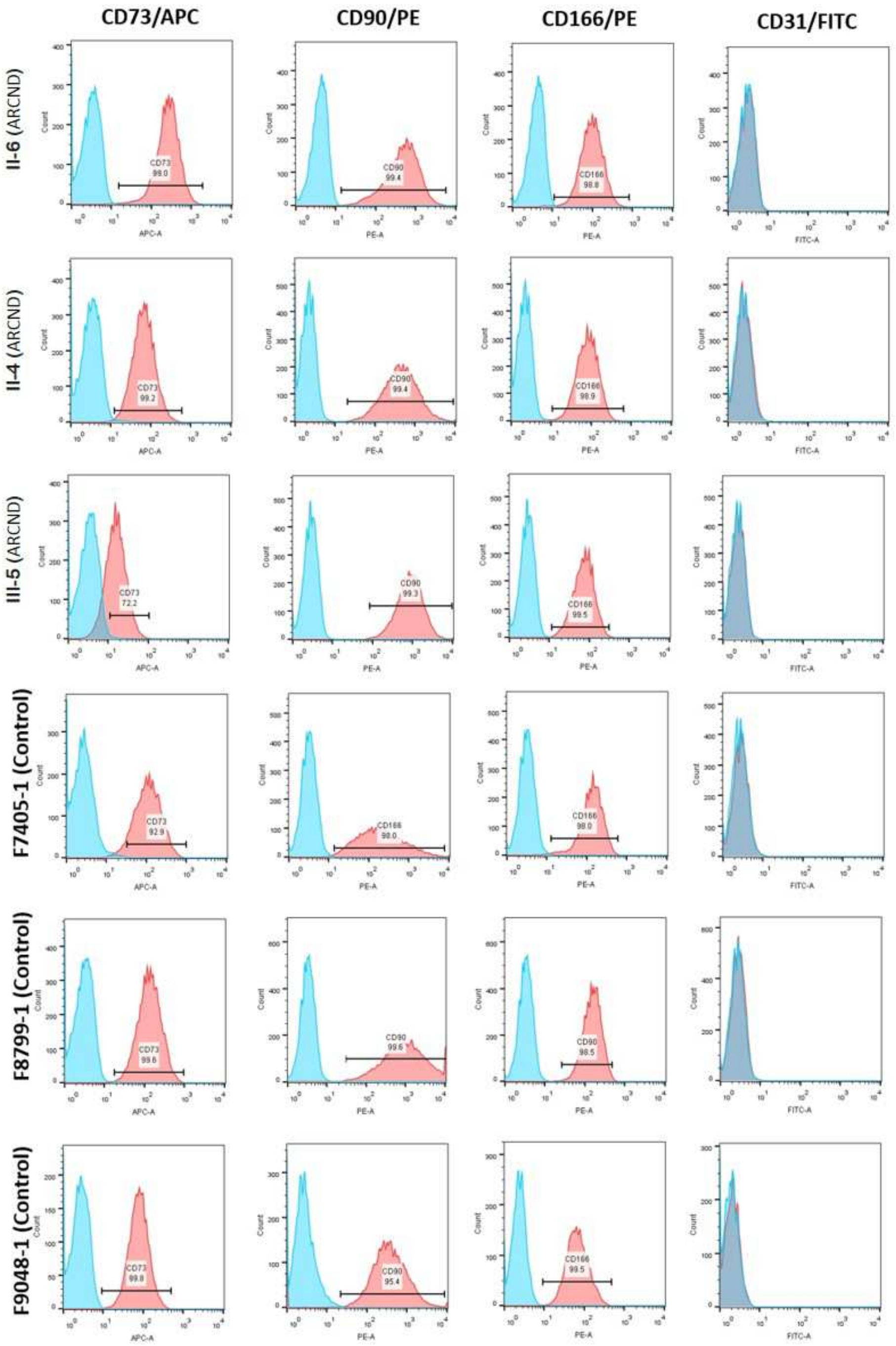

Supplementary Figure S8. Characterization of mesenchymal stem cells. Flow cytometry immunophenotype profile of MSCs showing positive staining for mesenchymal markers CD73, CD90 and CD166, and negative staining for endothelial marker CD31. Histograms represent event count (y-axis) vs. fluorescence (x-axis). Experimental data (red) were plotted in overlay with data from isotype controls (blue).

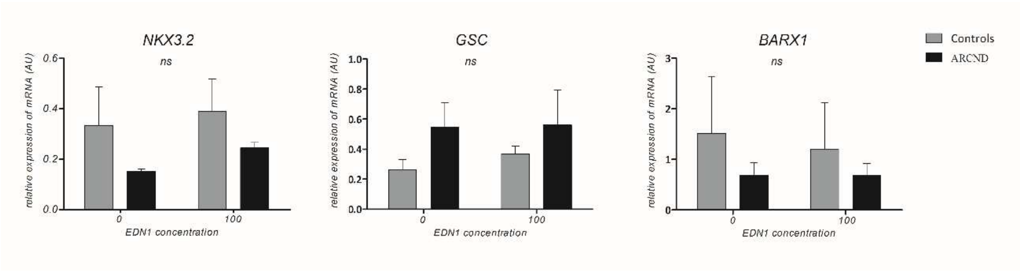

Supplementary Figure S9. mRNA expression analysis of mandible-related genes in NCC under EDN-1 treatment; not statistically significant differences were found in comparisons between controls and ARCND before and after EDN1 induction (two-way ANOVA). All values represent mean ± SEM.AU=arbitrary unit.

Romanelli Tavares VL, *et al. J Med Genet* 2022; 59:895–905. doi: 10.1136/jmedgenet-2021-107825
